# A Wearable Sensor-Based Exercise Biofeedback System: Mixed Methods Evaluation of *Formulift*

**DOI:** 10.2196/mhealth.8115

**Published:** 2018-01-31

**Authors:** Martin Aidan O'Reilly, Patrick Slevin, Tomas Ward, Brian Caulfield

**Affiliations:** ^1^ Insight Centre for Data Analytics University College Dublin Dublin Ireland; ^2^ School of Public Health, Physiotherapy and Sports Science University College Dublin Dublin Ireland; ^3^ Insight Centre for Data Analytics Dublin City University Dublin Ireland; ^4^ School of Computing Dublin City University Dublin Ireland

**Keywords:** mHealth, feedback, posture, exercise therapy, biomedical technology, lower extremity, physical therapy specialty

## Abstract

**Background:**

*Formulift* is a newly developed mobile health (mHealth) app that connects to a single inertial measurement unit (IMU) worn on the left thigh. The IMU captures users’ movements as they exercise, and the app analyzes the data to count repetitions in real time and classify users’ exercise technique. The app also offers feedback and guidance to users on exercising safely and effectively.

**Objective:**

The aim of this study was to assess the *Formulift* system with three different and realistic types of potential users (beginner gym-goers, experienced gym-goers, and qualified strength and conditioning [S&C] coaches) under a number of categories: (1) usability, (2) functionality, (3) the perceived impact of the system, and (4) the subjective quality of the system. It was also desired to discover suggestions for future improvements to the system.

**Methods:**

A total of 15 healthy volunteers participated (12 males; 3 females; age: 23.8 years [SD 1.80]; height: 1.79 m [SD 0.07], body mass: 78.4 kg [SD 9.6]). Five participants were beginner gym-goers, 5 were experienced gym-goers, and 5 were qualified and practicing S&C coaches. IMU data were first collected from each participant to create individualized exercise classifiers for them. They then completed a number of nonexercise-related tasks with the app. Following this, a workout was completed using the system, involving squats, deadlifts, lunges, and single-leg squats. Participants were then interviewed about their user experience and completed the System Usability Scale (SUS) and the user version of the Mobile Application Rating Scale (uMARS). Thematic analysis was completed on all interview transcripts, and survey results were analyzed.

**Results:**

Qualitative and quantitative analysis found the system has “good” to “excellent” usability. The system achieved a mean (SD) SUS usability score of 79.2 (8.8). Functionality was also deemed to be good, with many users reporting positively on the systems repetition counting, technique classification, and feedback. A number of bugs were found, and other suggested changes to the system were also made. The overall subjective quality of the app was good, with a median star rating of 4 out of 5 (interquartile range, IQR: 3-5). Participants also reported that the system would aid their technique, provide motivation, reassure them, and help them avoid injury.

**Conclusions:**

This study demonstrated an overall positive evaluation of *Formulift* in the categories of usability, functionality, perceived impact, and subjective quality. Users also suggested a number of changes for future iterations of the system. These findings are the first of their kind and show great promise for wearable sensor-based exercise biofeedback systems.

## Introduction

### Background

Resistance training is an exercise modality used in rehabilitation and strength and conditioning (S&C) settings. Adhering to a resistance training exercise program can increase a person’s muscular strength, hypertrophy, and power [1]. This can improve their sporting performance, mood, and quality of life [2,3]. However, many people completing exercise programs encounter various difficulties when performing their exercises without the supervision of a trained exercise professional such as an S&C coach. One such difficulty is that in such circumstances, people may execute their exercises incorrectly [4,5]. Incorrect alignment during exercise, incorrect speed of movement, and poor quality of movement may have an impact on the efficacy of exercise and may therefore result in a poor outcome [4,5]. Exercising with aberrant biomechanics may also heighten one’s risk of injury [6], necessitating technological solutions to provide accurate assessment of exercise form.

Inertial measurement units (IMUs) have been shown recently to be an accurate method for such exercise assessment. Wearable IMUs are able to acquire data pertaining to the linear and angular motion of limb segments and can also be used to measure a body’s three-dimensional orientation [7,8]. They are small, inexpensive, easy to set up, and facilitate the acquisition of human movement data in unconstrained environments [9]. Recent research has shown that a diverse range of exercises can be accurately evaluated with multiple and individual IMU setups [10-13]. These range from early stage rehabilitation exercises such as heel slides and straight leg raises [12] to more complex S&C exercises such as bodyweight squats [14], lunges [15], and single-leg squats [13,16,17]. More cost-effective and practical systems using a single body-worn IMU have also been shown to be effective in the analysis of exercise technique [13,15,18,19]. Such single IMU-based systems are considered preferential where they can provide equivalent exercise analysis quality to multiple IMU setups. Recently, it has been shown that for the detection of acute, naturally occurring deviations in compound lower-limb S&C exercises such as the barbell squat and the deadlift, personalized classification systems are superior in accuracy to the global ones [20]. It has also been shown that such personalized systems enable a single IMU to accurately classify repetitions of such exercises as “acceptable” or “aberrant” [20].

Although all the aforementioned work demonstrates the technological proficiency of IMU-based exercise biofeedback systems in classifying exercise technique, little is currently known about the user experience and users’ perceptions of such biofeedback systems. There is currently a surge of usability and system evaluation studies being published in the mobile health (mHealth) and ubiquitous health (uHealth) field [21-24]; however, there is a sparsity of such studies pertaining to IMU-based exercise biofeedback systems. Some past work has assessed the usability of IMU-based exercise biofeedback systems [25,26] but, to the author’s knowledge, there has not yet been any evaluation studies of biofeedback systems that classify exercise quality based on data from a wearable sensor and relay feedback to users via a mobile app.

**Figure 1:**
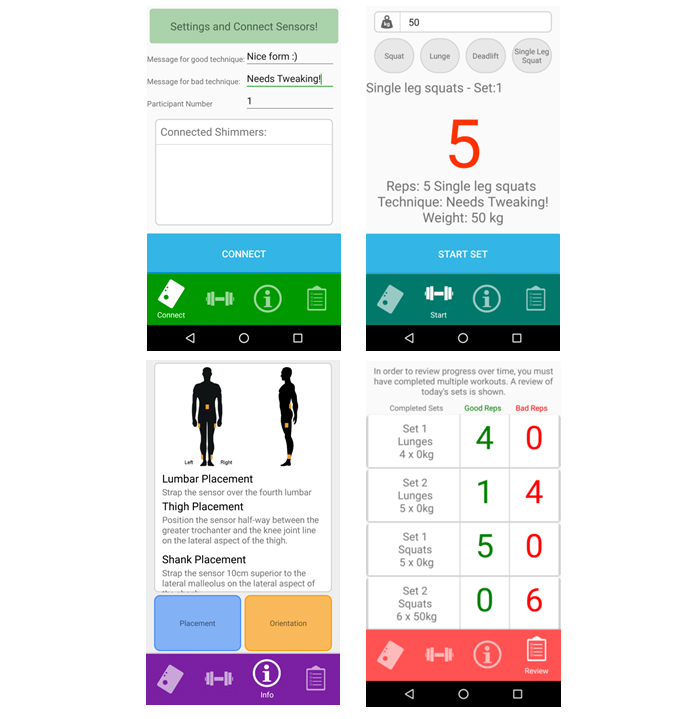
The *Formulift* app. User preferences and connecting to the inertial measurement unit (IMU; top left), real-time exercise biofeedback (top right), information and instructions (bottom left), and workout review (bottom right).

The study aimed to evaluate a recently developed IMU-based exercise biofeedback system called *Formulift.* *Formulift* consists of a mobile app and a single Shimmer IMU (Shimmer, Dublin, Ireland). The IMU is worn on the user’s left thigh and tracks their motion as they complete the following four exercises: squats, single-leg squats, lunges, and deadlifts. The mobile app processes the signals from the IMU, counts repetitions, and utilizes personalized classification methods to determine if each repetition completed of an exercise is “acceptable” or “aberrant.” During a set, the exerciser receives real-time feedback on the completion of repetitions of an exercise; this includes a vibration of the phone and an on-screen repetition counter. The user then receives feedback on their exercise technique following the completion of each set of an exercise. This feedback is shown in Figure 1 (top and bottom right) whereby after a set the exerciser is given a color-coded number indicating their technique quality and a message to reinforce this. The exerciser may then view how many repetitions were completed with acceptable and aberrant form on the review screen. The app also displays a pop-up message if two sets of the same exercise are completed sequentially with aberrant form. This message suggests they seek support of an exercise professional to identify and address their specific movement inefficiency. The app contains instructional information on how to do the exercises with acceptable technique and the option to review a workout session. However, these videos are not specific to the identified aberrant movements. A video comprehensively detailing the system can be seen in Multimedia Appendix 1. A number of screenshots from the app are shown in Figure 1.

### Objectives

The aim was to assess the system under a number of categories: (1) usability: the extent to which the system can be used by specified users to achieve specified goals with effectiveness, efficiency, and satisfaction in a specified context of use; (2) functionality: the ability of an interface or device to perform according to a specifically defined set of parameters [27] whereby the key functions of *Formulift* are to accurately detect and count repetitions of the exercises under study, determine if each repetition was completed with “acceptable” or “aberrant” technique, and provide the user with interpretable feedback on their completed exercise; (3) the perceived impact of the system; and (4) the subjective quality of the system. It was also desired to uncover suggested future improvements to the system. Three different realistic types of system users were employed to complete this evaluation: beginner gym-goers (<6 months experience), experienced gym-goers (>2 years’ experience), and qualified S&C coaches. Employing these three types of end users was hypothesized to enable a more comprehensive user-centered design approach to creating future iterations of the *Formulift* system.

## Methods

### Participants

A total of 15 healthy volunteers participated (12 males, 3 females, age: 23.8 years [standard deviation, SD 1.8], height: 1.79 m [SD 0.07], body mass: 78.4 kg [SD 9.6]). Group 1 included 5 beginner gym-goers with fewer than 6 months experience with resistance training and the exercises used in this study. Group 2 included 5 experienced gym-goers with a minimum of 2 years’ experience with resistance training and the exercises used in this study. The final group of system evaluators included practicing S&C coaches with qualifications from the National Strength and Conditioning Association or the United Kingdom Strength and Conditioning Association. Sample size numbers were chosen based on a combination of standard practice for completing qualitative usability studies [28] and in keeping with recent publications that also utilized the quantitative surveys used in this work [29,30]. No participant had a current or recent musculoskeletal injury that would impair their exercise performance. Each participant signed a consent form before completing the study. The University College Dublin Human Research Ethics Committee approved the study protocol.

### Data Collection

#### System Use

The testing protocol was explained to participants upon their arrival to the research laboratory. All participants completed a 5-min warm-up on an exercise bike; during which they were required to maintain a power output of 100 W and cadence of 75 to 85 revolutions per min. Following the warm-up, an investigator positioned a single IMU (Shimmer, Shimmer research, Dublin, Ireland) on the participant at the midpoint of the left femur (determined as halfway between the greater trochanter and lateral femoral condyle).

Video and IMU data were then simultaneously collected as the users completed the four following exercises: bodyweight left leg single-leg squats, bodyweight lunges, bodyweight or barbell squats, and barbell deadlifts. A total of 40 repetitions of each exercise were collected; 20 repetitions were completed with “acceptable” form, and 20 repetitions were completed with “aberrant” form. The “aberrant” repetitions from the 5 beginners were naturally occurring, whereas the 10 experienced participants deliberately induced their “aberrant’ form. Following this data collection, the IMU was removed from the participant’s left thigh.

The exercise professional then used the segmented videos to label each repetition of the four exercises as being “acceptable” or “aberrant” technique. Four binary random forests classifiers were then created for each participant, each pertaining to one of the four aforementioned exercises. These random forests objects were imported into the biofeedback app to make a personalized exercise classification system for each participant (Figure 1; Multimedia Appendix 1). While their personalized system was created, the participants completed a set of nonexercise-based tasks within the app. Multimedia Appendix 2 is the sheet given to participants listing the tasks that were to be completed “before the exercise analysis session.” They involved app navigation, interpretation of information within the app, and following instructions on system use and how to do the exercises. A full description of the exercise biofeedback system can be seen in the attached video (Multimedia Appendix 1).

Following the creation of their personalized biofeedback system, the participant first secured the IMU to their left thigh and then completed the list of tasks outlined in the “during exercise analysis section” of Multimedia Appendix 2. They first connected the wireless Shimmer IMU to the mobile app. They then completed two sets of ten repetitions for each of the four exercises. In the first set of each exercise, they were instructed to exercise with their best possible technique, and in the second, they were asked to try and replicate the mistake they had made before the exercise professional’s coaching. Throughout the session they were able to navigate to any point within the app including the “review tab” and to view any instructional content. The whole exercise session was observed by the investigator, who took note of any system crashes and the associated conditions, as the participant used the system and completed their exercises. The session was also simultaneously videoed for review following data collection. Upon completing the required exercises, participants were provided with the opportunity to test any other tasks within the app they desired. Participants then moved on to evaluating the system whereby they were administered surveys and partook in an interview.

#### Interviews

Immediately after completion of their exercise session with the system, a semistructured interview was conducted with each participant. A Dell Inspiron 5100 laptop (DELL, Texas, US) was used to video-record the interview. The webcam also captured the screen of the Android smartphone, allowing users to demonstrate any specific aspects of the app they wished to discuss. Each interview followed a topic guide to ensure consistent questioning across every interview [31]. This guide can be seen in Multimedia Appendix 3. Open-ended questions were used to garner participant’s views and experiences of the system in relation to usability, functionality, and perceived impact. Furthermore, participant’s reflections regarding their general evaluation of the system and suggested future changes were also captured.

#### Surveys

In addition to the interviews, the system was also assessed quantitatively utilizing two surveys. By mixing both quantitative and qualitative research and data, gains in breadth and depth of understanding and corroboration can be achieved, while offsetting the weaknesses inherent to using each approach separately.

The System Usability Scale (SUS) is a short, 10-point questionnaire that has been widely adopted in many domains as a fast and reliable measure of a system’s usability. The scale produces a usability score out of 100 (not a percentage) for every user who completes it. These scores can then be compared with the large body of published data on systems assessed with the SUS to find adjective and percentile rankings of a system’s usability [29,32].

The “user version of the Mobile Application Rating Scale” (uMARS) was also completed by all participants [22]. This is an adapted version of the “Mobile Application Rating Scale” and is more appropriate for end users of mobile apps [30]. It assesses the app under the areas of engagement, functionality, aesthetics, and information to produce an overall app quality score out of 5. The app’s subjective quality and perceived impact are also assessed separately. The perceived impact section of the survey was tailored to this study to investigate the app’s perceived impact on a person “exercising with their best technique.” No further adaptations were made to the uMARS survey for this work.

### Data Analysis

#### Qualitative

Interview recordings for all participants were transcribed verbatim and anonymized. A grounded-theory approach was then taken to the thematic analysis of the interview transcripts [33,34]. The interview topic guide was used to create an initial coding frame that was then refined as more data were analyzed. Data analysis was conducted by authors MOR and PS. Analysis involved scrutinizing the data to identify patterns, assigning codes to the data, and building themes and subthemes from the codes [35]. To maximize rigor and ensure the reliability of the theme extraction process, the researchers (MOR and PS) met frequently to evaluate the consistency of emergent themes and subthemes, which were further cross-checked for consistency across the three participant types (beginner gym-goers, experienced gym-goers, and S&C coaches) [36]. Discrepancies that were identified during these meetings were resolved through discussion between researchers, MOR and PS, until an agreement was reached. Data saturation was determined when no new data and no new themes and relationships among the interview data were emerging [37]. A table of themes and subthemes was created with associated quotes. This can be seen in Multimedia Appendix 4 of this paper.

#### Quantitative

The SUS score was computed for each participant following standard scoring methodology [38]. The mean and SD for the SUS scores was calculated for all participants and for each subgroup (beginner gym-goers, experienced gym-goers, and S&C coaches). The uMARS was also scored following standard procedures [22]. For each participant, a score out of 5 was calculated for engagement, functionality, aesthetics, and information. The mean of these four scores produced an “overall app quality score” for each app user. App subjective quality was quantitatively assessed taking each user’s star rating of the app. A perceived impact score, out of 5, was also found for each participant. The means and SDs of all the above scores were found for all participants and the three aforementioned subgroups.

## Results

### Summary

The *Formulift* system was assessed across four distinct domains: usability, functionality, perceived impact, and overall quality. In the upcoming subsections, results will be presented from both the quantitative surveys and qualitative interviews. Finally, suggested future changes will also be described.

### User Version of the Mobile App Rating Scale

The uMARS provided quantitative results on a number of key aspects of the app. A summary of results from the uMARS are summarized in Table 1. This table is referred to throughout the Results section.

### Usability and Functionality

The system achieved a mean SUS usability score of 79.2 (SD 8.8). Beginners deemed the system most usable with a score of 86.25 (SD 1.9), whereas the experienced gym-goers and S&C coaches scored the system at 75.5 (SD 9.1) and 74.5 (SD 8.0), respectively. These usability scores put the system at an 85% to 95% percentile based on all published research using the SUS [29] and would deem the system’s usability good to excellent on an adjective rating scale [32]. The functionality section of the uMARS also demonstrated an overall positive usability and functionality experience for users with a mean score of 4.2 (SD 0.37; Table 1). Although these surveys demonstrated that *Formulift* was deemed to have good usability, they provided limited insight in to the reasons for this and to what can be improved. This was found in analysis of the interview transcripts as described below.

Three key areas emerged from the interview data in relation to system usability: overall ease of use, the app’s interface, and the IMU. In terms of overall ease of use, 14 out of 15 participants remarked on the system being “easy to use,” “straightforward,” and “intuitive.” Example statements included the following:

I thought it was so easy to use...I just like how accessible it is as well.

Beginner gym-goer

Very, very easy to use. Really straightforward. You know, easy to get around and realise what you’re doing.

Experienced gym-goer

It’s very easy to navigate through. It’s pretty easy to be honest...It’s monkey see, monkey do really.

S&C coach

Many participants commented on the intuitive nature of the app. For instance, the layout of the app was acknowledged to be very easy to follow with large icons, large buttons and a minimal number of menus being cited as the reasons for this. Example statements included the following:

I mean, it’s quite user friendly. The interface; there’s not too much going on, on the screen. It’s very clear where the info tab is, where the exercise tab is etc.

S&C coach

Large buttons made that easy. It might come in to play more if you’ve got sweaty hands, but yeah in terms of navigation it was good. The size of the text and the buttons etc. is good. Overall, very good.

S&C coach

Yeah, the UI is really simple. Some other fitness apps are horrific. I hate using them, because they look horrible.

Beginner gym-goer

It was really easy to find things and navigate through.

Experienced gym-goer

None of the users reported any difficulties interpreting the language used within the app. The color used within the app was also referred to in a positive manner. It was considered to “make things stand out great” during a session, make the app “attractive,” and the color of the repetition number (red, orange, or green) following a set was said to be very useful:

I think the three color, “green, orange and red” feedback was a really useful function as it let you know if you’re doing something well, something a little bit off or doing something badly.

Beginner gym-goer

Participants also offered positive feedback regarding the “How to wear the sensor” section. They found the instructions were very clear and easy to follow:

One thing that I thought was done well was just showing you how to place the sensor as well. That could be a big obstacle, if it wasn’t shown properly. It would hinder people’s ability to use it. It was done well.

S&C coach

You go in to sensor placement/orientation you can’t go wrong there. If you do, you have an issue.

S&C coach

**Table 1:** Results from user version of the Mobile Application Rating Scale (uMARS) survey for beginner gym-goers, experienced gym-goers, and strength and conditioning (S&C) coaches. Overall quality is computed as described in the study by Stoyanov et al.

uMARS Section	Beginners (n=5) Mean (SD)	Experienced (n=5) Mean (SD)	S&C (n=5) Mean (SD)	All (n=15) Mean (SD)
Engagement	3.78 (0.48)	3.5 (0.42)	3.67 (0.33)	3.66 (0.42)
Functionality	4.27 (0.22)	4.24 (0.45)	4.08 (0.39)	4.2 (0.37)
Aesthetics	3.78 (0.50)	3.87 (0.81)	4.2 (0.34)	3.9 (0.62)
Information	4.29 (0.58)	4.2 (0.29)	3.9 (0.56)	4.14 (0.53)
Overall quality	4.03 (0.25)	3.95 (0.29)	3.96 (3.96)	3.98 (0.21)
Perceived impact	4.57 (0.39)	4.12 (0.45)	3.28 (0.52)	4.03 (0.70)
Star rating	3.83 (0.68)	4.0 (0.63)	3.6 (0.49)	3.8 (0.63)

In addition to this, a participant spoke positively about wearing the IMU:

I’m not conscience about wearing it, nobody can see it, it doesn’t feel weighty or anything like that. I almost forget it’s on my leg while I’m talking to you.

Beginner gym-goer

Participants reported a number of usability issues. The most reported usability issue related to app navigation, in particular, to going back a step within the app. Four participants, who are usually iPhone users, struggled initially to know how to navigate backwards in the app:

Maybe as I’m coming from IOS to Android but there was no clear back button so you have to switch in and out or use the phone’s button. On an iPhone, there’d always be something on the screen. That was one thing.

Beginner gym-goer

Just because I’m not used to using android, I didn’t know how to go back a step but other than that, no the app itself is very easy to navigate.

Experienced gym-goer

Some participants also highlighted the need for more status indicators as a usability issue. Particularly, they highlighted that within the “How to use the App” instructions, there was no on-screen indicator when one reached the last instruction, which meant they did not know that the final instruction had been reached. More importantly, the need for a loading indicator was highlighted when a user pressed the “Analyse my Set” button. One beginner gym-goer commented:

...when you don’t do that, I will impatiently tap the same button until something happens which in this case caused crashes.

Beginner gym-goer

In fact, this crash, caused by multiple taps of the “Analyse my Set” button was one of the most reported functionality issues. Although many users reported no bugs in the app, 5 users expressed experiencing a crash of the same manner. Two other critical bugs were found within the app that caused system crashes. The first was recorded by 4 users who reported a crash when quickly clicking through the “How to use the App” instructions:

There is a way of crashing it (the app). If you use the “how to use app menu” and go quickly through the menu, it’s pretty easy to crash. It seems like the second time it happens. You can scan through it the first time but not the second.

Beginner gym-goer

In the app instructions, I was tapping through quickly and it just crashed. I wasn’t mashing the button but I was pressing it reasonably quickly.

Experienced gym-goer

The second was experienced by 2 users who also found that the app crashed when they quickly navigated between the four main tabs of the app (connect, exercise, information, and review):

When I was exiting the app, when I had been looking at the exercises, it was just coming out and hitting all the buttons (demoes bashing all the menu buttons quickly) and the app crashed.

S&C coach

These were both programming bugs, which will be amended in future versions of the app. The most recurrent, nonfatal functionality issue mentioned by users regarded the real-time repetition counting during sets. Eight participants described thinking there was a lag at the start of the set, and after 2 or 3 repetitions, it was as if the app caught up and started counting them properly:

When I did the first rep of each set, I wasn’t sure if it was recording it, until I did the second rep. It would then say “2.” Sometimes it would take a couple seconds just to vibrate and register that I’d completed the repetition.

Beginner gym-goer

The rep counting was also a little bit slow at the start.

Experienced gym-goer

However, all participants felt the total repetition count was always correct. Participants also felt the binary classification of exercise repetitions (as acceptable or aberrant technique) was accurate. The beginners and experienced athletes found the system’s feedback useful. Gym-goers remarked the following:

It was really interesting how it could pick up on the bad ones and I know there were definitely some bad ones in there!

Beginner gym-goer

I usually am very aware of my form for sets but there was a set of single leg squats where I didn’t do the exercise well enough, and the app told me that I hadn’t, and I wasn’t aware of that but then when I thought about it the app was definitely right.

Experienced gym-goer

The S&C coaches, whose experience and knowledge allowed them to gain more insight to the accuracy of the system, were predominantly content with the system’s accuracy. Two S&C coaches did, however, feel the system misclassified a small number of specific repetitions:

It worked for everything except my single leg squats I’d say and maybe a little on the lunges.

S&C coach

Maybe one thing that it wasn’t able to discriminate on that well was the last set I did of shallow bodyweight squats. Maybe the accuracy fell off if I was doing something between a ¼ squat and a proper full squat. That was the only one that was a tiny bit inaccurate.

S&C coach

Overall, the SUS results, the uMARS, and the thematic analysis have demonstrated the system was usable and functional. The thematic analysis of the interview transcriptions has also uncovered a number of specific functionality issues and aspects of usability that can be amended or improved in future iterations of the app.

### Perceived Impact

The quantitative analysis of the perceived impact of the system, through the uMARS, demonstrated that the system was very beneficial to gym-goers in heightening their awareness of, advancing their knowledge of, increasing their motivation to, and their likelihood to seek help with “exercising with best possible technique” (Table 1). Thematic analysis of the interview transcriptions verified these quantitative findings and also uncovered a number of other perceived benefits and disadvantages to use of the system.

All users reported that using the system would aid their technique while exercising. Beginners often mentioned that the system would enable them to learn proper technique, whereas experienced gym-goers stated that the system would be useful particularly when they lose focus or increase the weight they are lifting, and S&C coaches thought the system would help people correct their technique and avoid injury while exercising. Statements included the following:

It’s also nice to have the feedback on how I’m actually doing things. Personally, when I go to the gym, I may even do a whole workout and not know if things have gone correctly. It’s pretty annoying to go home and be thinking, “Did I do my squats right today?,” “I’m not actually sure.”

Beginner gym-goer

For people who are just starting out with workout programs and need technique and form, it’s helpful. It’s helpful also for advanced weightlifting individuals who are looking to prevent injury and that kind of thing I would say.

Experienced gym-goer

A lot of the glaring issues people have when staring weight training are addressed. If people even just think about 1 or 2 of the issues that the app lays out then their technique can improve immensely in a very short amount of time just from these little bits of information.

S&C coach

Well advantages would be, obviously you’re avoiding injury as you go to the gym. This gives you a new source that can tell you if you’re doing it right or wrong or not.

S&C coach

Eight users also suggested that use of the system would have a positive effect on their focus and motivation to exercise with acceptable technique. S&C coaches also suggested that the system would be particularly useful in a team setting where athletes sometimes don’t process guidance properly or lose focus. All three test groups made statements regarding the system heightening focus, concentration, and/or motivation, such as:

It would be a motivational thing as well as obviously the benefit of getting help to correct yourself when you exercise poorly if needed.

Beginner gym-goer

Particularly, with me, when I’m sometimes doing weights I lose focus, so it would help me keep track.

Experienced gym-goer

You have definitely got some players where the information goes in one ear and straight out the other. So it would be good for us in the sense that we could connect this up, we analyze what we want to know and they find out straight away if they’ve done a good or bad rep.

S&C coach

Three out of six of the beginner gym-goers also spoke about the system as a tool to build their confidence in how they are exercising. They spoke of the app as a method to boost their likelihood of seeking help from a friend or trainer, a way to get over the initial anxiety of going to a gym, and to reassure them that they are exercising properly. One beginner spoke extensively of this, including saying:

Also, having something on my leg is really reassuring because I’ve always found that with fitness apps on my phone that direct me to exercise, I almost feel like all the information there can be interpreted wrong and when I go to do the exercises I might be misinterpreting them. But whatever it is, just having this on my leg just makes me feel a little bit more confident in doing them and interpreting the information that is provided by the app.

Beginner gym-goer

In addition to giving people confidence in their training, 3 S&C coaches felt one of the key benefits to the system is that it would boost people’s likelihood to simply start and commit to an exercise program. One of the S&C coaches in fact saw this as the biggest benefit to the system, and another spoke very positively of this aspect of system use:

The biggest benefit is it gets people in to the gym.

S&C coach

I think downloading the app could give a lot of people confidence to walk in to the gym in the first place, that’s really, really good.

S&C coach

The aforementioned subthemes of the perceived impact of system use, namely, (1) improving technique, (2) increasing motivation and focus, and (3) promoting participation in exercise, are all well accepted benefits of one having an S&C coach or personal trainer. Interestingly, a number of participants described the system either as a “virtual trainer” or a middle ground to having no personal trainer:

The app almost acts as a person telling you you’re doing it wrong. That’s how I felt.

Beginner gym-goer

I wouldn’t get a personal trainer but this system could be a good middle ground.

Beginner gym-goer

If you don’t want to hire a coach, as coaches are a lot of money then it will give you a pretty good overview of the kind of stuff you have to do.

S&C coach

There were no subthemes that emerged perceiving negative aspects to system use. However, one experienced gym-goer did suggest that use of the system could distract from focusing on their exercise technique. They stated the following:

It wasn’t necessarily confusing but I did think I might be paying more attention to the app than my own form.

Experienced gym-goer

### Subjective Quality

The subjective quality portion of the uMARS showed that, when available, 9 participants thought they would use this system 10 to 50 times over the next year, and 5 participants thought they would use it greater than 50 times in the next year. These 5 participants were all beginner gym-goers. All participants would recommend the system to people who might benefit from it. The median star rating from all 15 participants was 4 out of 5 (IQR: 3-4). The interview data reflected these quantitative ratings. All participants said they “liked” the system or thought it was “good.” More detailed statements included:

Overall, I was very impressed with the app. I have to say, very impressed.

Beginner gym-goer

I’ve been going to the gym for whatever amount of years and I’d still use something like this if it can tell me which reps are good and which reps are bad.

Experienced gym-goer

In terms of something to use during a session, I think it would be great.

S&C coach

In terms of aspects of quality to improve on, 2 S&C coaches felt the feedback was perhaps a little basic and could be more detailed:

Not that the technology it involves is, but in terms of how much information you could actually access it was quite basic.

S&C coach

One experienced gym-goer also expressed that without more feedback, they might stop using the system once they had perfected their technique:

I think the limit to the app is once you have the motion down, you’re less likely to keep using it.

Experienced gym-goer

Overall, all users subjective rating of system quality was positive. However, all participants had suggested improvements for future iterations of the app, which emerged as a theme during qualitative analysis and will be discussed in the upcoming subsection.

### Future Changes

The most popular suggestion for future changes to the system was to add more exercises that can be tracked. Beginners stressed the need for this, saying things such as:

I would like it if there were more exercises within the app as standard gym session would generally involve more exercises.

Beginner gym-goer

I think just add more exercises. Keep developing it as it’s just a great idea.

Beginner gym-goer

Maybe add some other type of movement that people do, I don’t know how well it transfers to upper body movements but certainly bench press is something that people always tend to need help with when they first go in to a gym.

S&C coach

I guess just add more exercises. So then it would cover more things, because I guess there is a wide range of exercises that people do when they go to the gym and they can all be done with poor form if you don’t know what you are doing.

S&C coach

Experienced gym-goers and S&C coaches regularly suggested the need for more exercises to be tracked by the system. However, because of their experience and knowledge, they also suggested the types of exercises that would require technique classification and suggested that for many exercises the system would only need to automatically count repetition and sets. There was a general consensus among the experienced gym-goers and S&C coaches that upper body compound exercises (eg, bench press, overhead press, and barbell row) were the additional exercises that should be incorporated to the system, including technique classification. There was also a shared opinion that users should be able to add any exercise they complete to the system to be logged automatically. However, it was suggested that noncompound, secondary exercises may not require technique classification, as they are associated with a lower injury risk. It was also said that Olympic weightlifting moves should not be added to the battery of assessed movements, as they would be too dangerous to learn via an app. Two statements that summarize the cohort’s general opinion are as follows:

Because, it’s in an app; I would say prioritize...you could have compound exercises like a bench press or an OHP (over-head press) but like you can’t teach like a jerk or a clean so just compound or isolation movements as there is less that can go wrong with those kind of things.

S&C coach

In terms of other exercises; again I suppose I like the idea that it would manly be your key lifts. In terms of adding loads of other exercises, I don’t know if it would be necessary. The ones we would mainly cover in terms of injury risk are your squat, your back squat, your deadlift etc. So yeah, in terms of that I’d keep it to key lifts.

S&C coach

Feedback was another prominent subtheme that emerged in the area of future changes. Two key things were suggested recurrently: (1) providing longitudinal feedback or progress over time and (2) more detailed feedback on the completed exercise repetitions. With regards to providing longitudinal feedback, one beginner suggested the following:

I’d be really interested to use it regularly and see if I look back over weeks am I seeing progress?...(Comparison to SleepTracker) So I’d like to see something similar in this where you could link your exercise quality to your habits and progress.

Beginner gym-goer

This type of longitudinal feedback reflected what most users of the system would also desire. With regards to receiving more specific feedback on their completed exercise, a diverse range of suggestions were made:

Then after the end of your sets, if it tells you like an estimate of your maximum and could count your rest times.

Beginner gym-goer

It would be really interesting to actually see the angles.

Experienced gym-goer

A drop down with exactly what reps are good and bad would be useful.

Experienced gym-goer

We’d be quite keen on muscle fiber recruitment during an exercise. I’m not sure if the sensors can pick up on it.

S&C coach

Tempo—that would be a big one for us.

S&C coach

The most frequently reported request, however, was to receive feedback on the exact mistake one was making when exercising, as opposed to whether a repetition was simply “good” or “bad”:

Maybe, for example, “your back was too arched” or “not arched enough,” or the angle of your legs, how far down you should be going etc.

Experienced gym-goer

When participants mentioned this, they were informed of other work from the authors that uses multiple IMUs to classify the exact deviation one makes while completing the exercises [14,15,39]. They were then asked if they would rather prefer a multi-IMU system that may be more expensive than the evaluated single IMU system if it could identify specific exercise mistakes. Opinions were mixed on this, with 2 participants stating it “would depend on cost.” However, one beginner, one experienced gym-goer, and one S&C coach did suggest they would actually prefer a multi-IMU system which had such capability:

I do actually think more sensors would be cool but I think, I think that because I’m a bit of a nerd with stuff, so I’m like more sensors, that’s cool; more accurate data etc. I think for the people you may actually be selling this app to, one sensor is actually nearly too much.

Beginner gym-goer

I think I’d like more sensors and feedback. Wearing sensors doesn’t put me off.

Experienced gym-goer

I suppose, because I’m dealing with high level athletes, I would prefer to have more sensors to get more information.

S&C coach

No other subthemes regarding future changes were found; however, one S&C coach did suggest a team version of the system, where multiple users could connect to a coach’s tablet app. This would allow them to focus their time and attention to the team members who require it the most. They also suggested the straps and IMU should be improved to be more appealing in such a setting.

## Discussion

### Principal Findings

To the authors’ knowledge, this study is the first to apply a mixed-methods approach to evaluating a wearable sensor-based exercise biofeedback system. In particular, this study is a first look at users’ perceptions of such systems and their potential benefits. Therefore, in addition to providing information on the usability, functionality, and perceived impact of *Formulift*, the presented results also offer a number of end-user insights that can be leveraged to inform the development of future exercise biofeedback systems.

#### Usability and Functionality

The results demonstrated a good to excellent overall level of system usability. Participants highlighted that the *Formulift* system was easy to set up and intuitive in nature, particularly in relation to the ease at which they could complete tasks. This shows great promise for the uptake of wearable sensor-based exercise biofeedback apps within beginner and experienced gym-goer populations. However, analysis of the data also uncovered a number of specific usability issues that will be amended in future iterations of the app. For instance, the app should incorporate more status indicators, for example, the appearance of a loading screen while exercise data are being analyzed through the “Analyse my set” function. This addition would signal to the user that an action is taking place, thus reducing the user's uncertainty related to the completion of the task. A clearer method for navigating backwards in the app should also be added. Such changes should minimize confusion for system users and enable a more enjoyable and efficient user experience.

This study has also demonstrated that the *Formulift* system is functional. The key desired functions of *Formulift* are to accurately detect and count repetitions of the exercises under study, determine if each repetition was completed with “acceptable” or “aberrant” technique, and provide the user with interpretable feedback on their completed exercise. The combination of both qualitative and quantitative results shows that the system was indeed functional in these three areas. This was not withstanding a number of functionality bugs that were found during the study. The most significant bug was the real-time repetition counting algorithm lagging at the start of some user’s sets. This must be rectified for future iterations of the system. It is essential that the real-time repetition counting functions correctly because if it does not, it may distract the user from completing their exercise properly.

#### Perceived Impact

One of the most important findings of this study is that the range of different system users (beginner gym-goers, experienced gym-goers, and S&C coaches) reported several benefits to using the *Formulift* system. Most importantly, all users felt that the system would improve their technique as they exercise. This is a central finding as prior work has simply shown the ability of IMU-based exercise classification systems to detect “acceptable” and “aberrant” technique but has not determined if users would find feedback of this kind beneficial [12,15,17,39-41]. Interestingly, users also highlighted the system’s positive psychological benefits with regards to improved levels of focus, motivation, and confidence while exercising. These perceived benefits are in line with desired aims for such systems as outlined in prior research [12,15,17,39-41]. Although these benefits are well reported aims for many biofeedback systems [42], the literature currently lacked end-user validation. Further study is required to objectively validate these perceived benefits. Despite no negative impacts of *Formulift*’s perceived impact emerging as subthemes, one participant did point out that the phone’s position during exercise (ie, in the user's hand or on the floor in front of them) may be distracting from proper technique. This matter is not yet fully understood, and future iterations of the system should factor in how the phone is positioned during exercise to maximize the system’s benefit to the user.

#### Subjective Quality

En masse, *Formulift* was well received by system users. The uMARS results showed the app had a median star rating of 4 out of 5. This shows that users thought the system was good but could also be improved. This feeling was backed up during participants’ interviews. Although suggested improvements to the system will later be discussed, it is an important finding of this study that system users did like *Formulift*. Wearable sensor-based exercise biofeedback systems are a very new technology, and little is yet known about how users feel about using them. Therefore, it is encouraging to developers of such systems that the participants of this study gave predominantly positive feedback on the system.

### Future Work

The systematic evaluation of the *Formulift* system uncovered a variety of suggested changes for future system iterations. Future work will endeavor to incorporate these changes in to the system or allow them to be customizable user preferences within the system, for example, turning on or off receiving feedback on tempo of movement. Additionally, future work will aim to establish users’ perceptions of the system following multiple uses over a period of time. Such work would also grant the opportunity to (1) Objectively measure the impact of the system for users in a more rigorous manner and (2) Investigate how these findings compare to the system’s perceived impact found in this study.

### Comparison With Prior Work

As stated in the introduction to this paper, a vast proportion of the published work pertaining to exercise analysis with IMUs regards the efficacy of the various sensor setups and data analysis techniques to assess different exercises [10-19]. However, while in the early development phase of such systems, it is also of key importance to understand their usability, functionality, subjective quality, and perceived impact from the end user perspective. Involving the user early and often in the design process can help identify previously unforeseen user experience issues that can then be rectified to help increase levels of user engagement which is a central determinant to overall user adoption prospects [43].

To the author’s knowledge, there are no published evaluation studies of wearable sensor-based exercise biofeedback systems. Literature is available in associated fields, with a vast variety of mHealth and exergaming systems being evaluated over the past decade [24,44]. It may be inappropriate to compare the results evaluating *Formulift* to apps and systems in other subdisciplines in mHealth because of the different demographics of users and purposes of such systems. However, it is important to note that the methodological approach undertaken in this study is in line with current state-of-the-art recommendations in usability studies [24]. Involving three types of real system users (beginners, experienced, and S&C) and employing a mixed-methods approach to evaluate the system has maximized our understanding on users’ perceptions of *Formulift* and will inform the design of future iterations. Although recent work such as that by Kotsantinidis et al who outlined the development and evaluation of “FitForAll”: an Exergaming Platform Improving Physical Fitness and Life Quality of Senior Citizens [45] has shown great benefits to exercise biofeedback systems and demonstrated good SUS scores, a lack of qualitative assessment of the system limits the conclusions that can be drawn on the system’s usability and user experience. It is the authors’ contention that we can maximize the learnings on mHealth system evaluations through combining appropriate surveys and interviews. This approach can more effectively inform the iterative design process to make the systems as beneficial as possible for end users.

### Limitations

There are a number of contextual factors that should be considered when reviewing this study. To begin with, all results presented in this study are based on the participants’ first use of the system. Although this is likely appropriate for highlighting any usability and functionality problems with the system, it is possible that one’s rating of the system’s quality and impact could vary over time. It should also be stated that results regarding the “perceived impact” of the system, as determined by the uMARS and thematic analysis of interview transcripts, are solely users’ opinions on the benefits of the system. More work is required to determine if the system objectively improves, for example, people’s motivation, exercise adherence, and exercise technique. To achieve this, a longitudinal study will be required. It should also be noted that this study took place in an artificial gym environment within a biomechanics laboratory. It would be useful to complete the proposed longitudinal system evaluation with participants exercising in their “normal” gyms. This may help uncover additional usability or functionality issues that should be amended and other future changes which could improve the system. A further limitation of this study is the sample size used and the homogeneity of the sample. While a sample of 15 participants complies with usability testing standards, it may not guarantee that the sample is representative of the wider population when considering quantitative results. This may be the case in particular for specific populations not captured in our sample, for example, elderly, overweight, or underweight. However, there was high consistency when triangulating quantitative and qualitative results, which suggests they are both of merit. It should also be noted that this paper was concerned with determining the usability, functionality, perceived impact, and subjective quality of the *Formulift* system, and as such, expansive detail has not been provided on the following topics: the exact data analysis pathways utilized within the *Formulift* app; the quantitative performance of the system during the evaluation (ie, system accuracy, sensitivity, and specificity); and the manner by which the experienced exercisers and S&C coaches completed deliberately “aberrant” movements. These aspects of the system evaluation experiment are detailed in a recent paper by the authors (MOR, TW, and BC) [46]. This associated paper also details a tablet app that aims to ameliorate the overhead in setting up personalized classifiers for each exerciser, that is, the following processes are streamlined: synchronizing video and IMU data collection, signal processing, data segmentation, data labeling of segmented videos by an exercise professional, feature computation, and classifier creation [46]. Limiting the work involved in creating personalized exercise classification systems for new system users may be a key factor for their uptake in to the real world.

### Conclusions

In this study, we sought to evaluate the *Formulift* system, a new exercise biofeedback app that classifies technique and tracks repetitions completed of exercises. A mixed-methods approach was undertaken to quantitatively and qualitatively assess the system under a variety of distinct categories: usability, functionality, subjective quality, and perceived impact. The assessment of the system was completed by three types of real system users: beginner gym-goers, experienced gym-goers, and qualified and practicing S&C coaches. The usability of the system was determined to be very good following both quantitative and qualitative analysis. The system also functioned as desired with users reporting that the system accurately detected their repetitions in real time, classified their exercise quality, and gave them appropriate feedback. Users expressed that they liked the system and that it could aid their focus and technique while exercising. Additionally, it was found that the system could increase their motivation and confidence in completing exercise. These findings are the first of their kind and show great promise for wearable sensor-based exercise biofeedback systems. However, this study also found a great deal of potential improvements to the *Formulift* system (Future Work subsection). By implementing these changes, it is hoped that systems such as *Formulift* may become an affordable, user-friendly, and useful tool that will aid gym-goers to enhance their training and support S&C coaches in the monitoring of their athletes.

## Appendix

Multimedia Appendix 1 Video highlighting the *Formulift* system features and typical use case.

Multimedia Appendix 2 List of tasks presented to users for system evaluation session.

Multimedia Appendix 3 Interview guide used for qualitative assessment of the *Formulift* system.

Multimedia Appendix 4 Table of themes arising from thematic analysis of interview transcripts.

## Abbreviations

IMU: inertial measurement unit
IQR: interquartile range
mHealth: mobile health
S&C: strength and conditioning
SUS: system usability scale
uHealth: ubiquitous health
uMARS: user version of the Mobile Application Rating Scale
