# Supplementary material for: A Wearable Sensor-Based Exercise Biofeedback System: Mixed Methods Evaluation of Formulift
Source: JMIR Mhealth Uhealth. 2018 Jan 31;6(1):e33. doi: 10.2196/mhealth.8115 (PMC5812980; doi:10.2196/mhealth.8115)
Supplement: Multimedia Appendix 2 [file mhealth_v6i1e33_app2.pdf]

App Evaluation Study – List of Tasks – Participant _____

Before Exercise Analysis Session

Open the ‘Formulift’ app.

Find and view information about how to position and wear the sensor.

Find and view information on how to use the app to analyse your exercise technique.

Navigate through app to the various screens detailed in the instructions.

Find and view the information videos about the four following exercises: Squat, Lunge, Deadlift and Single Leg Squat.

During Exercise Analysis Session

Open the ‘Formulift’ app.

Input the messages you would like to receive when you complete an exercise with good/bad form/technique.

Connect the smartphone to the Bluetooth sensor.

Navigate to the exercise analysis section of the app.

Select the ‘squat’ exercise and input the weight you are going to lift.

Press the ‘Start Workout’ button when you are in position to and ready to start a set.

When you are ready complete the following sequence of exercises, feel free to view the ‘review tab’ or ‘info tab’ between sets of exercises.

Exercise	Reps	Weight (kg)	Rest time (s)	Technique Style
Squats	10			Best possible form
Squats	10			
Left leg forward lunges	10			Best possible form
Left leg forward lunges	10			
Deadlift	10			Best possible form
Deadlift	10			
Left leg single leg squats	10			Best possible form

View the review tab and the information available there.

Complete any other tasks within the app and when happy, close the app.
