# Supplementary material for: A Wearable Sensor-Based Exercise Biofeedback System: Mixed Methods Evaluation of Formulift
Source: JMIR Mhealth Uhealth. 2018 Jan 31;6(1):e33. doi: 10.2196/mhealth.8115 (PMC5812980; doi:10.2196/mhealth.8115)
Supplement: Multimedia Appendix 3 [file mhealth_v6i1e33_app3.pdf]

Formulift: Interview Guide

Context

- Q 1. How experienced are you in the exercises you completed today?
  - Q 1.1. How many years have you done for them?
- Q 2. How technologically proficient are you?
  - Q 2.1. Are you usually an iPhone or an Android user?
  - Q 2.3. Do you use other health and fitness apps?

Overall experience

- Q 3. What did you think of the app?
  - Q 3.1. Why?
  - Q 3.2. How did formulift compare to other health and fitness apps you use?

Usability

- Q 4. How did you find completing tasks while using the app?
- Q 5. What did you think of navigation/scrolling/colour/font size/language?

Functionality

- Q 6. Did you think there were any bugs in the app?
- Q 7. Do you think the system works?

Perceived Impact

- Q 8. What do you think the benefits or disadvantages to using the app are?
  - Q 8.1. Why?

Closing remarks

- Q 9. Is there anything else you would like to say about the app?
- Q 10. What other things would you like to see in future versions of the app?
