# Supplementary material for: A Wearable Sensor-Based Exercise Biofeedback System: Mixed Methods Evaluation of Formulift
Source: JMIR Mhealth Uhealth. 2018 Jan 31;6(1):e33. doi: 10.2196/mhealth.8115 (PMC5812980; doi:10.2196/mhealth.8115)
Supplement: Multimedia Appendix 4 [file mhealth_v6i1e33_app4.pdf]

## Multimedia Appendix 4 – Table of Themes

| **Theme:**<br>**Usability and functionality** | **Supporting Evidence** | **Participant: Beginner (B), Experienced (E), or S&C Coach (S&C)** |
|---|---|---|
| **Sub-Theme:**<br>*Navigation:* Need for a back button on screen | *Things like, if I go in to how to use the app, if I want to go back I have to hit the back button, I can't just hit the logo button to go back. For me it's quicker to just use the tab button.* | B |
| | *I think when I was going through the videos as well. I had to tap out of the current video item to see another one. So I had to click on the toolbar, to go to the page where it counts your reps and then go back. So it was little counterintuitive thing. There could be a button to just navigate out from the video section but straight back to the info tab.* | B |
| | *Just because I'm not used to using android, I didn't know how to go back a step but other than that, no the app itself is very easy to navigate.* | B |
| | *Maybe as I'm coming from IOS to Android but there was no clear back button so you have to switch in and out or use the phone's button. On an iPhone, there'd always be something on the screen. That was one thing.* | E |
| *Interface:* Need for status indicators. | *I think the main reason for that is when I pressed buttons, particularly those ones, there is no status indicator. Maybe, just if there was a colour change or a slight indicator just to show that my action has been registered within the app. because when you don't do that, I will impatiently tap the same button until something happens which in this case caused crashes.* | B |
| | *It was when you got to the end of this (instructions), there was no clear indication that you had finished all the instructions, so just get rid of the arrow or something* | E |
| *Interface:* Colour | *But I think the three colour, 'green, orange and red' feedback was a really useful function as it let you know if you're doing something well, something a little bit off or doing something badly.* | B |
| | *The colour indication is good.* | E |
| | *No, so navigating through the app, I found very easy. I found the different colours to make different things stand out great. Like I said, during a session it would be very quick and easy to navigate through.* | S&C |
| | *Good colour. It's bright, it's colourful, it's attractive.* | S&C |
| *Functionality:* rep counting | *It was pretty accurate. Every so often there'd be an extra vibration. I don't know if it was measuring an extra one. At one point I thought it did measure an extra squat but then it didn't increment the next time I actually did a squat.* | B |
| | *I did, I felt the buzzes happened at the right time and that the rep count was correct at the end of the set. It was fine, because once you got used to it you knew the reps would tally up to the correct number but it was just a bit weird that when you start going to actually do the reps there could temporarily be a wrong number displayed. It was just a small thing, just freaked me out a little bit.*<br>*I thought sometimes it could be quite sensitive. I don't know, when I was going through some of my reps and things like that, I found that sometimes it would count a rep early for me, even when I was just literally starting.* | B |
| | *When I did the first rep of each set, I wasn't sure if it was recording it, until I did the second rep. It would then say '2'. Sometimes it would take a couple seconds just to vibrate and register that I'd completed the repetition.* | E |
| | *When you start your set the rep count doesn't start from zero every time. It might start sometimes at 2 and then it kind of catches up.* | E |
| | *The rep counting was also a little bit slow at the start.* | E |
| | *Yes, each rep was counted. Obviously, there might be a small delay but every* | S&C |

| | | |
|---|---|---|
| | *rep was counted accurately.* | |
| *Functionality:* Technique classification accuracy | *It was really interesting how it could pick up on the bad ones and I know there were definitely some bad ones in there!* | B |
| | *I usually am very aware of my form for sets but there was a set of single leg squats where I didn't do the exercise well enough, and the app told me that I hadn't  and I wasn't aware of that but then when I thought about it the app was definitely right.* | E |
| | *The accuracy was good as well.* | S&C |
| | *Maybe one thing that it wasn't able to discriminate on that well was the last set I did of shallow bodyweight squats. Maybe the accuracy fell off if I was doing something between a ¼ squat and a proper full squat. That was the only one that was a tiny bit inaccurate.* | S&C |
| | *It picked up really well on the deadlift stuff, the knee valgus in the lunge and single leg squats also.* | S&C |
| | *It worked for everything except my single leg squats I'd say and maybe a little on the lunges.* | S&C |
| *Usability:* Sensor and strap | You can't disconnect the sensor but I guess that might be on purpose. | B |
| | *How to wear the sensor?....fine.* | B |
| | *There was just one thing regarding the orientation of the sensor. It was described really well but the way it's shown in the app, is not the same way it looks when I look down at it on my leg, instead it's like when it's down on the table in front of me, that's how it's depicted in the app but once I do strap it on, I'm looking at it and just trying to work out is that the same as the app says? I did figure it out, but it's just a thing that could have made it a little smoother for me.* | B |
| | *One thing that I thought was done well was just showing you how to place the sensor as well. That could be a big obstacle, if it wasn't shown properly. It would hinder people's ability to use it. It was done well.* | S&C |
| | *It's good in that, I'm not conscience about wearing it, nobody can see it, it doesn't feel weighty or anything like that. I almost forget it's on my leg while I'm talking to you.* | B |
| | *The sensor, seeing as you only have one of them would be quite easy to just strap on to your leg, assuming you wouldn't need tape or anything, that would be a disadvantage.* | S&C |
| | *It's not cumbersome to set up currently but I think the strap should be improved.* | S&C |
| | *In terms of the instructions on how to actually set yourself up and strap the sensor on to your leg, I had to read it a couple of times but I don't see how it could be any more clear. I think it's just that I had to read it a couple of times because it's just a foreign thing to me currently.* | S&C |
| | *Setting up with this, placement etc. doesn't seem to difficult but if you were to have a number of people, it might be difficult.* | S&C |
| | *You go in to sensor placement/orientation you can't go wrong there. If you do, you have an issue.* | S&C |
| **Perceived impact of system use** | | |
| Impact on user's technique | *It's also nice to have the feedback on how I'm actually doing things. Personally, when I go to the gym, I may even do a whole workout and not know if things have gone correctly. It's pretty annoying to go home and be thinking, 'Did I do my squats right today?', 'I'm not actually sure'.* | B |
| | *I'd feel like there is a lot lower risk of injury because if you're doing things wrong you're going to injure yourself quite quickly.* | B |
| | *I think it would help in my technique. I suppose you become more conscience of how you're doing something due to the feedback it gives you between the sets.  An outcome of using this app would definitely be better technique.* | B |
| | *You are getting the benefit of technique correction I suppose, with the app* | B |

| | | |
|---|---|---|
| | *as it is.* | |
| | *For people who are just starting out with workout programmes and need technique and form, it's helpful. It's helpful also for advanced weightlifting individuals who are looking to prevent injury and that kind of thing I would say.* | E |
| | *I think it would actually benefit more experienced lifters/athletes more. They could basically just check that they are maintaining good technique all the time. Because if the app tells them they were wrong, they are more likely to be able to know the mistake they've been making so you just want to avoid it and track that improvement* | E |
| | *If you got this to somebody that was learning this exercises, I think it would be fantastic. They'll learn on a rep by rep basis if they're doing the correct motion.* | E |
| | *It would improve my technique.* | E |
| | *The benefits would be being able to go to a normal gym and actually keep an eye on your technique.* | B |
| | *It would be a motivational thing as well as obviously the benefit of getting help to correct yourself when you exercise poorly if needed.* | B |
| | *So the benefits to the user would be that they have a technique corrector if they were doing this work on their own.* | S&C |
| | *A lot of the glaring issues people have when staring weight training are addressed. If people even just think about 1 or 2 of the issues that the app lays out then their technique can improve immensely in a very short amount of time, just from these little bits of information.* | S&C |
| | *Well advantages would be, obviously you're avoiding injury as you go to the gym. This gives you a new source that can tell you if you're doing it right or wrong or not.* | |
| Focus/Concentration /Motivation | *Yeah, I think so; I think it makes you concentrate a lot more on what you're doing, as opposed to just going along with it.* | B |
| | *Particularly, with me, when I'm sometimes doing weights I lose focus, so it would help me keep track.* | E |
| | *It's like a motivational thing, when you get feedback it's easier to do it right.* | B |
| | *It would be a motivational thing as well as obviously the benefit of getting help to correct yourself when you exercise poorly if needed* | B |
| | *I also think, in terms of athlete questioning you could say 'were there any reps there that felt bad?' then compare that to the feedback that you get, rep by rep. Something like that, it might be a good indication to ask your athlete 'well why do you think that might have been bad? What do you think it picked up on?' That would open a nice area for questioning there.* | S&C |
| | *You have definitely got some players where the information goes in one ear and straight out the other. So it would be good for us in the sense that we could connect this up, we analyse what we want to know and they find out straight away if they've done a good or bad rep. So they can go, if they see they've done 30/40% poor form, they have to come see us and talk about it.* | S&C |
| Reassurance/ Confidence | I guess a lot of people would be too shy to go directly to somebody for advice or maybe even just to take the advice from somebody who is willing to give it to them. Whereas, having it here on an app is quite a private way to get feedback. | S&C |
| | It could help me get over that initial anxiety that you have. | B |
| | The app would build people's confidence. | B |
| | Also, having something on my leg is really reassuring because I've always found that fitness apps on my phone that direct me to exercise, I almost feel like all the information there can be interpreted wrong and when I go to do the exercises I might be misinterpreting them. But whatever it is, just having this on my leg just makes me feel a little bit more confident in doing them and interpreting the information that is provided by the app.- | B |

| | | |
|---|---|---|
| | Some times in the gym you can feel self-conscience so you don't necessarily want to be doing squats in front of a full body mirror. So having an app that tells me that my squats are wrong might just give me that kick up the ass to think, I actually need to look at myself and do this properly.- | B |
| Efficiency | *Benefits to this are, especially in the team setting, would be efficiency to look at everybody all at once. Obviously there it's quite hard to keep an eye on 10-15 people at once. So if you had a bit of feedback on that.* | S&C |
| Participation | *The biggest benefit is it gets people in to the gym.* | S&C |
| | *I think downloading the app could give a lot of people confidence to walk in to the gym in the first place, that's really, really good.* | S&C |
| Formulift as a virtual coach/Guidance | *The app almost acts as a person telling you you're doing it wrong. That's how I felt.* | B |
| | *I felt a benefit was having the directed types of exercise and having the videos there. I didn't have to like go search for a video or whatever.* | B |
| | *It would allow be people to just start with say the bar and then safely progress to adding weight. Even for people who have been using the gym for a long time, there are always things you can be improving on.* | B |
| | *I wouldn't get a personal trainer but this system could be a good middle ground* | B |
| | *If you don't want to hire a coach, as coaches are a lot of money then it will give you a pretty good overview of the kind of stuff you have to do.* | S&C |
| **Future Changes to Formulift** | | |
| Tracking more exercises | *I assume these aren't the only 4 you're going to work with and that you'll expand.* | B |
| | *I think just add more exercises. Keep developing it as it's just a great idea.* | B |
| | *I would like it if there were more exercises within the app as standard gym session would generally involve more exercises.* | B |
| | *Even though this worked really well, I think it could be built in to more of just a tracking type app. If it was, when I went to the gym I would just use this app for everything, technique checking and tracking. I think that would be good.* | E |
| | *It would be cool to have more exercises but a way that you can log your normal workout session and then that it tracks your continuous progress in some way.* | E |
| | *Maybe some other type of movement that people do, I don't know how well it transfers to upper body movements but certainly bench press is something that people always tend to need help with when they first go in to a gym.* | S&C |
| | *I guess just add more exercises. So then it would cover more things, because I guess there is a wide range of exercises that people do when they go to the gym and they can all be done with poor form if you don't know what you are doing.* | S&C |
| | *I would definitely put in more exercises. If you had like a full battery of bodyweight exercises or exercises you can do in the gym, that would be enormously helpful to everybody.* | S&C |
| | *Some sort of upper body protocol.* | S&C |
| | *In terms of other exercises; again I suppose I like the idea that it would manly be your key lifts. In terms of adding loads of other exercises, I don't know if it would be necessary. The ones we would mainly cover in terms of injury risk are your squat, your back squat, your deadlift etc. So yeah, in terms of that I'd keep it to key lifts.* | S&C |
| Additional Feedback | *The bodyspace thing also give you rest times as well.* | B |
| | *Something like a feed of sets would be helpful enough.* | B |
| | *Then after the end of your sets, it tells you like an estimate of your maximum.* | B |
| | *Like little details like being able to make your own.* | B |

| | | |
|---|---|---|
| | *It would be really interesting to actually see the angles.* | E |
| | *If it had specific comments, maybe, for example, 'your back was too arched' or 'not arched enough' , or the angle of your legs, how far down you should be going.* | E |
| | *A drop down with exactly what reps are good and bad would be useful.* | E |
| | *More specific feedback, like the exact mistake you are making.* | E |
| | *I think seeing it across several variables. So rather than just good technique and bad technique.* | S&C |
| | *We'd be quite keen on muscle fibre recruitment during an exercise. I'm not sure if the sensors can pick up on it.* | S&C |
| | *Tempo, that would be a big one for us. And maybe centre of mass, or my centre line for the limb it's worn on.* | S&C |
| | *All 3 of them (ECC/AMOR/CON). When we lift, it's quite important for the eccentric phase, especially if we're doing a box squat or a squat, the rule is 3-4 seconds on the way down and that's just to help resilience and injury prevention. But you do notice when the guys are younger versus more experienced, they don't understand it. They rush down too quick and they actually skip the whole phase like. So if we could show them good time and good tempo to control the bar and control the weight, they could get a lot better and it would be definitely useful.* | S&C |
| Wearing more sensors to enable more detailed feedback | *I do actually think more sensors would be cool but I think, I think that because I'm a bit of a nerd with stuff, so I'm like more sensors, that's cool; more accurate data, bla bla bla. I think for the people you may actually be selling this app to, one sensor is actually nearly too much.* | B |
| | (multi-label system) Maybe, that would be a premium package or whatever. | E |
| | I think I'd like more sensors and feedback. Wearing sensors doesn't put me off. | E |
| | (multi-label system) I would probably, this is me personally, but I would look at the cost set up, is it monthly or is it a once off? If it was pay more for the sensors but then the monthly cost was the same as one sensor, then I would maybe go for that. | B |
| | (multi-label system)I suppose, because I'm dealing with high level athletes, I would prefer to have more sensors to get more information. For example, maybe one on the upper body or different sensors there. | S&C |
